# An economic evaluation of knee osteoarthritis treatments in Thailand

**DOI:** 10.3389/fphar.2022.926431

**Published:** 2022-09-26

**Authors:** Parnnaphat Luksameesate, Aree Tanavalee, Suthira Taychakhoonavudh

**Affiliations:** ^1^ Department of Social and Administrative Pharmacy, Faculty of Pharmaceutical Sciences, Chulalongkorn University, Bangkok, Thailand; ^2^ Department of Orthopaedics, Faculty of Medicine, Chulalongkorn University, Bangkok, Thailand

**Keywords:** knee osteoarthritis, economic evaluation, cost-utility analysis, glucosamine, etoricoxib

## Abstract

**Objective:** The objective of this study is to evaluate the cost-effectiveness of different knee OA care sequences compared to standard treatment reimbursed by the major health insurance payer in Thailand.

**Method:** We used decision analytical modeling to evaluate the effect of either adding etoricoxib or crystalline glucosamine sulfate compared to standard treatment from a societal perspective over patients’ lifetimes. Data were analyzed based on efficacy, whereas adverse events were considered as a substate. Model input data were retrieved from relevant published literature and the Standard Cost Lists for Health Technology Assessment, Thailand. All health outcomes were measured in a unit of quality-adjusted life-year (QALY). An incremental cost-effectiveness ratio (ICER) was applied to examine the costs and QALYs. Sensitivity analysis was performed to investigate the robustness of the model.

**Result:** The results demonstrated that adding crystalline glucosamine sulfate (before diclofenac plus proton pump inhibitors, PPI) into the standard care sequence was a dominant strategy compared to the standard care sequence. Adding etoricoxib alone or including crystalline glucosamine sulfate (after diclofenac plus PPI) was dominated by adding crystalline glucosamine sulfate (before diclofenac plus PPI), whereas in a willingness-to-pay (WTP) threshold in Thailand, adding of both crystalline glucosamine sulfate (before diclofenac plus PPI) and etoricoxib were cost-effective when compared to adding crystalline glucosamine sulfate alone with ICER of 125,547 Thai baht/QALY (3,472 US dollars/QALY).

**Conclusion:** The addition of crystalline glucosamine sulfate and etoricoxib into standard knee OA treatment were cost-effective at the WTP threshold in Thailand. In addition, early initiation of crystalline glucosamine sulfate would be less costly and more effective than delayed treatment or the use of standard treatment alone.

## Introduction

Osteoarthritis (OA), the most common chronic condition of the joints, is the deterioration of the cartilage in joints resulting in bones rubbing together and leading cause of pain, stiffness, swelling, and disability. The global prevalence of knee OA was 22.9% in people of 40 years and over (Cui et al., 2020), whereas in Thailand, the prevalence of knee OA in 2018 was approximately 8.64% (Department of Thai Traditional and Alternative Medicine, 2019). The number of knee OA patients have been gradually increasing as the aging society becomes an issue globally. The economic burden of knee OA is also high in not only direct medical costs associated with OA but also indirect costs (Centers for Disease Control Prevention (CDC), 2007; Murphy and Helmick, 2012; Hatoum et al., 2014).

The treatment for OA is broadly divided into five groups: acetaminophen, oral non-steroidal anti-inflammatory drugs (NSAIDs), symptomatic slow-acting drugs for osteoarthritis (SYSADOA), intra-articular steroid injection, and surgery (Royal College of Orthopedic Surgeons of Thailand (RCOST), 2011). The most common treatment for knee OA is the use of NSAIDs because of its efficacy for pain relief. Nevertheless, the risk of developing NSAID‐related gastrointestinal and cardiovascular complications are concerned (Mosler, 2014). Glucosamine sulfate, a SYSADOA treatment, is one of an alternative treatments for mild to moderate knee OA. Glucosamine has a very good safety profile; however, its efficacy is still an arguable issue resulted in differences in regulatory status as well as the reimbursement of the product (Luksameesate and Taychakhoonavudh, 2021).

In Thailand, the reimbursement policy of knee OA treatments among three major health insurance schemes, namely, Civil Servant Medical Benefit Scheme (CSMBS), Universal Coverage Scheme (UC), and Social Security Scheme (SSS), varies as some medicines are being reimbursed by one health insurance payers but not the others. For example, glucosamine is reimbursable with some restrictions under CSMBS but is not reimbursed under UC and SSS (Tantivess and Tangcharoensathien, 2016).

Several economic evaluation studies of knee OA treatment were conducted to compare NSAIDs either selective COX-2 NSAIDs or non-selective NSAIDs (Yen et al., 2004; Contreras-Hernández et al., 2008) as well as glucosamine sulfate (Black et al., 2009; Chaiyakunapruk et al., 2010; Scholtissen et al., 2010; Bruyère et al., 2019). However, most of these studies compared between two knee OA treatments and focused only on efficacy or adverse events but not on both aspects of knee OA treatment together. The issue of knee OA treatments become complex as it is not a question of choosing the most effective or cost-effective treatment over the other but rather the decision to incorporate the treatment into the care sequence in which the efficacy is to delayed progression with the optimal tolerable side effects of the treatment itself. To our understanding, no research has been done to systematically evaluate knee OA treatment in which all OA treatments including acetaminophen, NSAIDs, SYSADOA, intra-articular corticosteroid, and total knee arthroplasty (TKA) are being compared altogether. The objective of this study is to evaluate the cost-effectiveness of different knee OA care sequences compared to standard treatment reimbursed by the major health insurance payer in Thailand from a societal perspective.

## Materials and methods

A cost-utility analysis was conducted to compare among treatment options for knee OA patients aged 45 or over with mild to moderate pain and no comorbidities. All health outcomes were measured in a unit of quality-adjusted life-year (QALY). We analyzed based on the standard treatment while considering the adverse events of all care sequences. Model input data were obtained from the Drug and Medical Supply Information Center (DMSIC), Ministry of Public Health (MoPH) and relevant published literature. The analysis was done from a societal perspective. Costs and QALYs were each discounted at 3% as recommended by the Guideline for Health Technology Assessment (Guideline Development Working Group, 2019). The willingness-to-pay threshold in Thailand was determined as 160,000 Thai baht (THB)/QALY (Chaikledkaew and Teerawattananon, 2013). The cycle of the study was 6 months and a lifetime horizon.

### Model structure

A Markov model was used in our decision analytical modeling. The core concept of three previous literatures were applied to our Markov model structure which incorporates all knee OA treatments and its adverse events (Losina et al., 2013; Capel et al., 2014; Katz et al., 2016). The standard care sequence was based on the clinical guideline of knee OA treatment published by the Royal College of Orthopedic Surgeons of Thailand (RCOST) 2011 (Royal College of Orthopedic Surgeons of Thailand (RCOST), 2011), available evidence, and experts’ opinions. Either etoricoxib or crystalline glucosamine sulfate or both were incorporated into the standard care sequence as an intervention to compare the results of delaying disease progression and to calculate cost-effectiveness.

**FIGURE 1:**
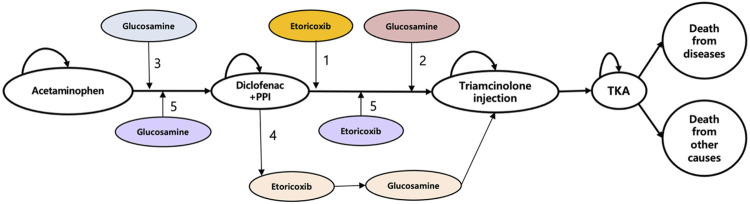
Model structure. PPI, proton pump inhibitors; TKA, total knee arthroplasty.

In this study, five treatment states were used as a standard treatment to model OA prognosis based on the knee OA treatments including acetaminophen, diclofenac plus a proton pump inhibitors (PPI), triamcinolone acetonide (TA) injection, total knee arthroplasty (TKA), and death (Figure 1). A total of five alternatives of either etoricoxib or crystalline glucosamine sulfate or both were added into standard treatment to compare with the standard treatment alone as follows:

1) the addition of etoricoxib;
2) the addition of crystalline glucosamine sulfate (after diclofenac plus PPI state);
3) the addition of crystalline glucosamine sulfate (before diclofenac plus PPI state);
4) the addition of crystalline glucosamine sulfate (after diclofenac plus PPI state) and etoricoxib;
5) the addition of crystalline glucosamine sulfate (before diclofenac plus PPI state) and etoricoxib.

We also took adverse events into account as sub-state including gastrointestinal (GI) discomfort, symptomatic ulcer, myocardial infarction (MI), heart failure (HF), and stroke.

In each cycle of the model, patients could move either to the next progressive state where advanced treatment is needed or the death state (absorbing state) according to the transition probabilities.

An incremental cost-effectiveness ratio (ICER) was applied to examine the costs and QALYs of knee OA treatments. All patients in the study began the model from the initial “acetaminophen” state to the “death” state and transition probabilities were used for each cycle of the model.

### Model assumptions

The main assumption of our study was that patients received only assigned treatment in those states and the risks of adverse events were constant over time. Transition to the next treatment option was also irreversible.

Regarding the literature review of economic evaluation of knee osteoarthritis, all studies including our study use the Markov model in which either sequence of treatment or adverse events are designed as model states. This is due to the nature of osteoarthritis disease in which disease status is hard to identify (Bessette et al., 2009; Latimer et al., 2009; Brereton et al., 2012; Losina et al., 2013; Capel et al., 2014; Katz et al., 2016; Karasawa et al., 2021). The treatment of osteoarthritis thus usually depends on the pain, symptoms, and activities of daily living of patients. If the pain persists, treatment will be changed to higher potency and thus next model states. Therefore, we assume that treatment failure in which the patient move to the next state is when the patient reports the persistence of pain. The subject whose pain was not relieved on those states was failure resulting in discontinuation and the subject would move to the next treatment state.

### Model inputs

A structured literature review was conducted to explore relevant published studies in Thailand. Where local data were unavailable, data from the randomized controlled trial (RCT), meta-analysis, systematic review, and real-world evidence studies were considered. Base-case probability estimates and ranges over a period of 6 months are presented in Table 1, and the model parameter is shown in Table 2.

**TABLE 1:** Base-case probability estimates and ranges over a period of 6 months.

Drug	Pain relief	GI discomfort	Symptomatic ulcer	Stroke	MI	Heart failure	Sources of evidence
Acetaminophen	0.3380	0.2382	0.0007	0.0005	0.0013	0.0000	Herrero‐Beaumont et al. (2007)
							Latimer et al. (2011)
Diclofenac + PPI	0.8647	0.1013	0.0044	0.1012	0.2289	0.0007	Zacher et al. (2003)
							Cannon et al. (2006)
Etoricoxib	0.5657	0.0556	0.0014	0.1071	0.2107	0.0010	Reginster et al. (2007)
							Cannon et al. (2006)
Crystalline glucosamine sulfate	0.3857	0.1093	0.0000	0.0019	0.0019	0.0000	Herrero‐Beaumont et al. (2007)
							Sawitzke et al. (2010)
TA injection	0.0392	0.0000	0.0000	0.0000	0.0000	0.0000	McAlindon et al. (2017)
TKA	0.5122	0.0000	0.0899	0.0000	0.0469	0.0100	Fortin et al. (1999)
							Husted et al. (2010)
							Bullock et al. (2003)
							Bohm et al. (2012)

GI, gastrointestinal; MI, myocardial infarction; PPI, proton pump inhibitors; TA, triamcinolone acetonide; TKA, total knee arthroplasty.

**TABLE 2:** Model parameter.

Component	Estimate	SE	Sources of evidence
Costs of treatment; THB (USD)			
Acetaminophen (3,000 mg/day)	213 (6)	-	Drug And Medical Supply Information Center MoPH (2018)
Diclofenac (150 mg/day)	128 (4)	-	Drug And Medical Supply Information Center MoPH (2018)
Omeprazole (20 mg/day)	94 (3)	-	Drug And Medical Supply Information Center MoPH (2018)
Etoricoxib (60 mg/day)	5,213 (144)	-	Drug And Medical Supply Information Center MoPH (2018)
Crystalline glucosamine sulfate (1,500 mg/day)	1874 (52)	-	Drug And Medical Supply Information Center MoPH (2018)
TA injection (40 mg every 3 months)	114 (3)	-	Drug And Medical Supply Information Center MoPH (2018)
Total Knee Arthroplasty (TKA)	78,925 (2,183)	-	Vivattanavarang (2011)
Treatment cost-related adverse events; THB (USD)			
GI discomfort	436 (12)	11 (0.31)	Limwattananon et al. (2005)
Symptomatic ulcer	3,734 (103)	95 (3)	Limwattananon et al. (2005)
Stroke in the 6 months	56,133 (1,552)	1,432 (40)	Tamteeranon et al. (2007)
Stroke in the first year	7,173 (198)	183 (5)	Tamteeranon et al. (2007)
Stroke in the subsequent year	10,029 (277)	256 (7)	Tamteeranon et al. (2007)
Myocardial infarction in the 6 months	138,916 (3,842)	3,544 (98)	Anukoolsawat et al. (2006)
Myocardial infarction in the first year	4,706 (130)	120 (3)	Anukoolsawat et al. (2006)
Myocardial infarction in the subsequent year	13,588 (376)	347 (10)	Anukoolsawat et al. (2006)
Heart failure in the 6 months	15,347 (424)	392 (11)	Permpanicha et al. (2015)
Heart failure in the first year	3,974 (110)	101 (3)	Permpanicha et al. (2015)
Heart failure in the subsequent year	7,948 (220)	203 (6)	Permpanicha et al. (2015)
Indirect costs; THB (USD)			
Travel cost	143 (4)	12 (0.33)	The Health Intervention and Technology Assessment Program (HITAP) (2017)
Food cost	53 (1)	5 (0.14)	The Health Intervention and Technology Assessment Program (HITAP) (2017)
Utilities of knee OA pain			
Moderate Pain	0.56	0.0152	Wajajamroen (2016)
No pain	0.62	0.0036	Wajajamroen (2016)
Disutilities of adverse events			
GI discomfort	−0.0228	0.0001	Sullivan and Ghushchyan (2006)
Symptomatic ulcer	−0.0269	0.0002	Sullivan and Ghushchyan (2006)
Stroke	−0.0524	0.0001	Sullivan and Ghushchyan (2006)
Myocardial infarction	−0.0409	0.0002	Sullivan and Ghushchyan (2006)
Heart failure	−0.0635	0.0002	Sullivan and Ghushchyan (2006)

SE, standard error; TA, triamcinolone acetonide; GI, gastrointestinal; OA, osteoarthritis; DMSIC, MoPH, Drug and Medical Supply Information Center, Ministry of Public Health; HITAP, The Health Intervention and Technology Assessment Program; THB, Thai baht; USD, US dollars.

### Efficacy of pain relief

All efficacy data were identified by RCT studies worldwide due to limited efficacy data available in Thailand (Fortin et al., 1999; Zacher et al., 2003; Herrero‐Beaumont et al., 2007; Reginster et al., 2007; McAlindon et al., 2017). The inclusion criteria of selected studies were knee OA patients who had most relevant characteristic to the Thai population and received the treatment. Nevertheless, the treatment or daily dose which was differently defined from our study was excluded. Crystalline glucosamine sulfate was the sole formulation included in the study for estimation. The efficacy of pain relief for each treatment state was derived from the outcome measurement of the Western Ontario and McMaster Universities Osteoarthritis Index (WOMAC) pain score, a self-administered health status assessing pain for patients with painful arthritis of the knee. Efficacy was then converted to probability for use in the model.

### Adverse events

Adverse events of acetaminophen were retrieved from the cost-effectiveness study (Latimer et al., 2009), whereas large RCT studies estimated adverse events of diclofenac, etoricoxib, glucosamine, and TA injection (Cannon et al., 2006; Sawitzke et al., 2010; McAlindon et al., 2017). A total of three obsevational study were used to extracted each adverse event of TKA (Bullock et al., 2003; Husted et al., 2010; Bohm et al., 2012).

To deal with the heterogeneity of multiple data sources, we applied the most appropriate method to parameterize this model as a base-case analysis, and sensitivity analysis was performed for the remaining data.

### Costs

For the base-case analysis, direct and indirect costs were included in the study. Direct medical costs consisted of the cost of therapies, cost of treatments due to adverse events, laboratory cost, outpatient visit cost, inpatient visit cost, and doctor fees. Direct non-medical costs consisted of traveling costs and additional food costs. Indirect costs included productivity loss due to pain. All cost data were converted at the rate of 36.16 THB/US dollars (USD) (Bank of Thailand, 2022).

Costs of the drugs in this model were calculated by assuming that patients received treatment with the maximum dose for knee OA treatments (Thai Rheumatism Association, 2010; Wells et al., 2015). The hospital purchasing prices that were available from the Drug and Medical Supply Information Centre of the Ministry of Public Health were used to calculate drug cost (Drug And Medical Supply Information Center MoPH, 2018). Data from the generic drug cost data were used for the base-case analysis, and the original drug cost was used for the sensitivity analysis. Cost of TKA was obtained from the database of Chiangrai Prachanukroh Hospital from October 2009 to September 2011 (Vivattanavarang, 2011). Costs of treatment for adverse events including GI discomfort, symptomatic ulcer, stroke, MI, and HF were obtained from the cost-effectiveness study in Thailand.

Direct non-medical costs consisted of traveling costs and additional food costs. Indirect costs included productivity loss due to pain. The data was retrieved from the Standard Cost Lists for Health Technology Assessment, Thailand (The Health Intervention and Technology Assessment Program (HITAP), 2017).

### Utilities

The utility of each health state was measured by the efficacy of the drug to relieve pain. Patients who moved to the next treatment regimen due to uncontrollable pain were given a utility of 0.56 in the previous health state since the medication given could not control the OA symptoms. The utility data, one local data availability, were retrieved from the data collected by Wajajamroen (2016) using EQ-5D-3L index scores for knee OA patients at Nopparat Rajathanee Hospital, Thailand. Since no disutility of data was done in Thailand, the disutilities of adverse events including GI events and Cardiovascular (CV) events from Sullivan and Ghushchyan (2006) were collected using preference-based EQ-5D index scores for chronic conditions in the United States.

### Sensitivity analyses

A sensitivity analysis was performed to investigate the robustness of the model. A deterministic sensitivity analysis (DSA) was used to examine the model’s results when the changed value in the specific input parameters. The key parameters were selected from the literature including the cost of crystalline glucosamine sulfate, cost of TKA, transition probability of diclofenac plus PPI, transition probability of TA injection, transition probability of TKA, and utility of knee OA pain. A probabilistic sensitivity analysis (PSA) was performed using Monte Carlo simulation. The 1,000 iterations generated a different value of cost and QALYs, which were demonstrated by the incremental cost-effectiveness plane and cost-effectiveness acceptability curve (CEAC). Costs were underlying gamma distributions. Probabilities and utilities were underlying beta distributions. Transition probabilities were underlying log-normal distributions.

## Results

### Base-case analysis

The base-case analysis was done in patients of 45 years or over with mild to moderate pain and no comorbidities. The results demonstrated that adding crystalline glucosamine sulfate (before diclofenac plus PPI state) into the standard care sequence was a dominant strategy compared to the standard care sequence. Adding etoricoxib alone or including crystalline glucosamine sulfate (after diclofenac plus PPI state) was dominated by adding crystalline glucosamine sulfate (before diclofenac plus PPI). When comparing the rest of the alternatives we found that adding of both crystalline glucosamine sulfate (before diclofenac plus PPI state) and etoricoxib incurred a higher total cost [366,819 THB (10,144 USD) vs. 150,878 THB (5,325 USD)] and gained more QALYs (4.98 vs. 3.26) than adding crystalline glucosamine sulfate alone. This yielded an ICER of 125,547 THB/QALY (3,472 USD/QALY) and was cost-effective under the willingness-to-pay (WTP) threshold in Thailand (160,000 THB/QALY). The total costs, total QALYs, and ICER in six treatment alternatives under base-case conditions are given in detail in Table 3.

**TABLE 3:** Base-case results.

Treatment alternative		Cost; THB (USD)	QALYs	ICER; THB (USD) per QALY
1	Standard treatment	161,282 (4,460)_	2.39	
2	Standard treatment + Glucosamine (before diclofenac plus PPI)	150,878 (4,173)	3.26	Dominant
3	Standard treatment + Glucosamine (after diclofenac plus PPI)	192,561 (5,325)	3.26	Dominated
4	Standard treatment + Etoricoxib	418,268 (11,504)	4.44	Dominated
5	Standard treatment + Etoricoxib + Glucosamine (before diclofenac plus PPI)	366,819 (10,144)	4.98	125,547 (3,472)
6	Standard treatment + Etoricoxib + Glucosamine (after diclofenac plus PPI)	431,478 (11,932)	4.98	Dominated

PPI, proton pump inhibitors; QALYs, quality-adjusted life-years; ICER, incremental cost-effectiveness ratio; THB, Thai baht; USD, US dollars.

### Sensitivity analyses

**FIGURE 2:**
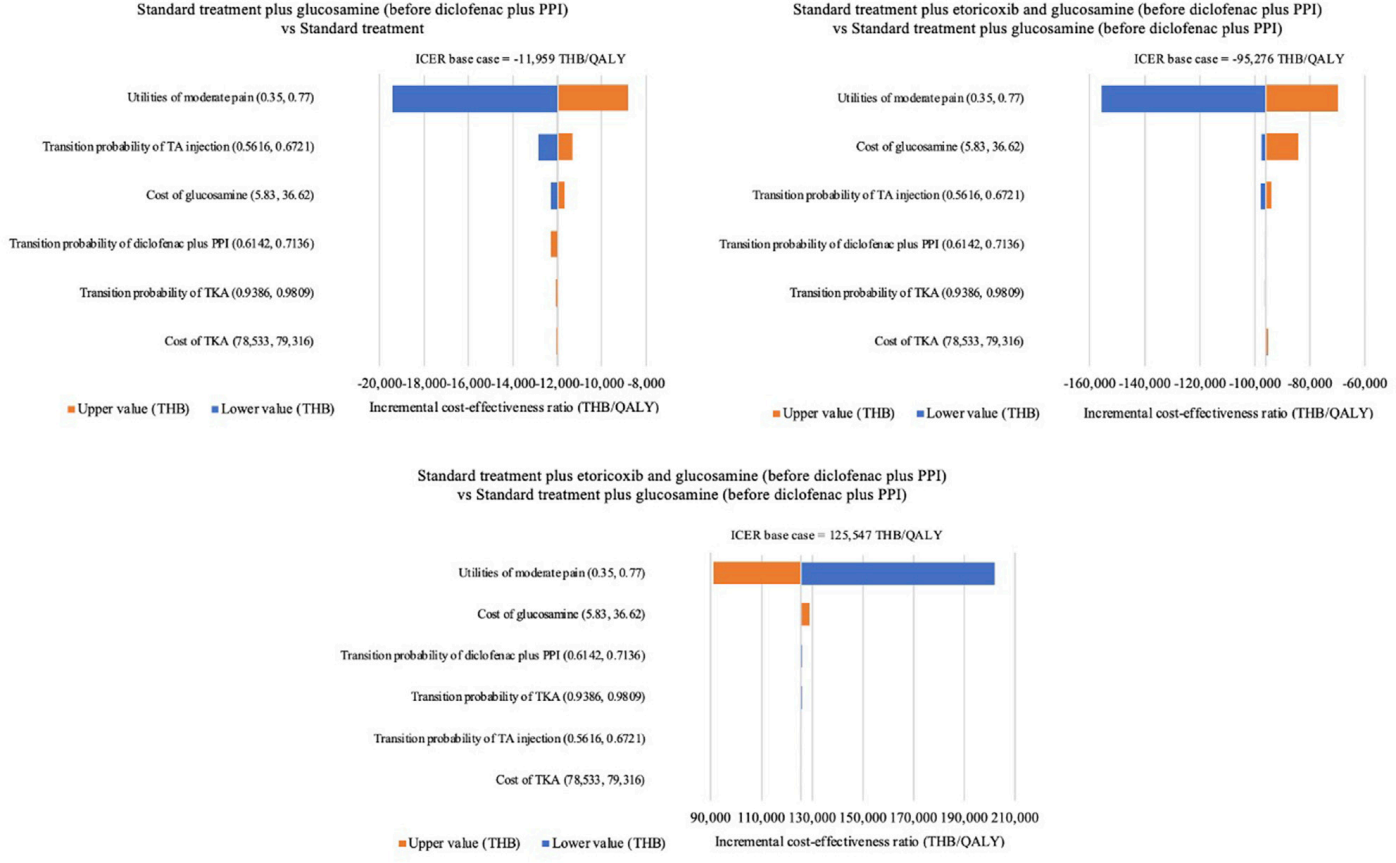
Tornado diagram. PPI, proton pump inhibitors; TA, triamcinolone acetonide; TKA, total knee arthroplasty; QALYs, quality-adjusted life-years; THB, Thai Baht.

Based on the one-way sensitivity analysis, the effect of the utility of moderate pain had the highest impact of all six alternative results when varying values between 0.35 and 0.77. The cost-effectiveness ratio was less sensitive to the cost of TKA results when varying values between 78,533 THB (2,172 USD) and 79,316 THB (2,193 USD). The tornado diagram is presented in Figure 2.

**FIGURE 3:**
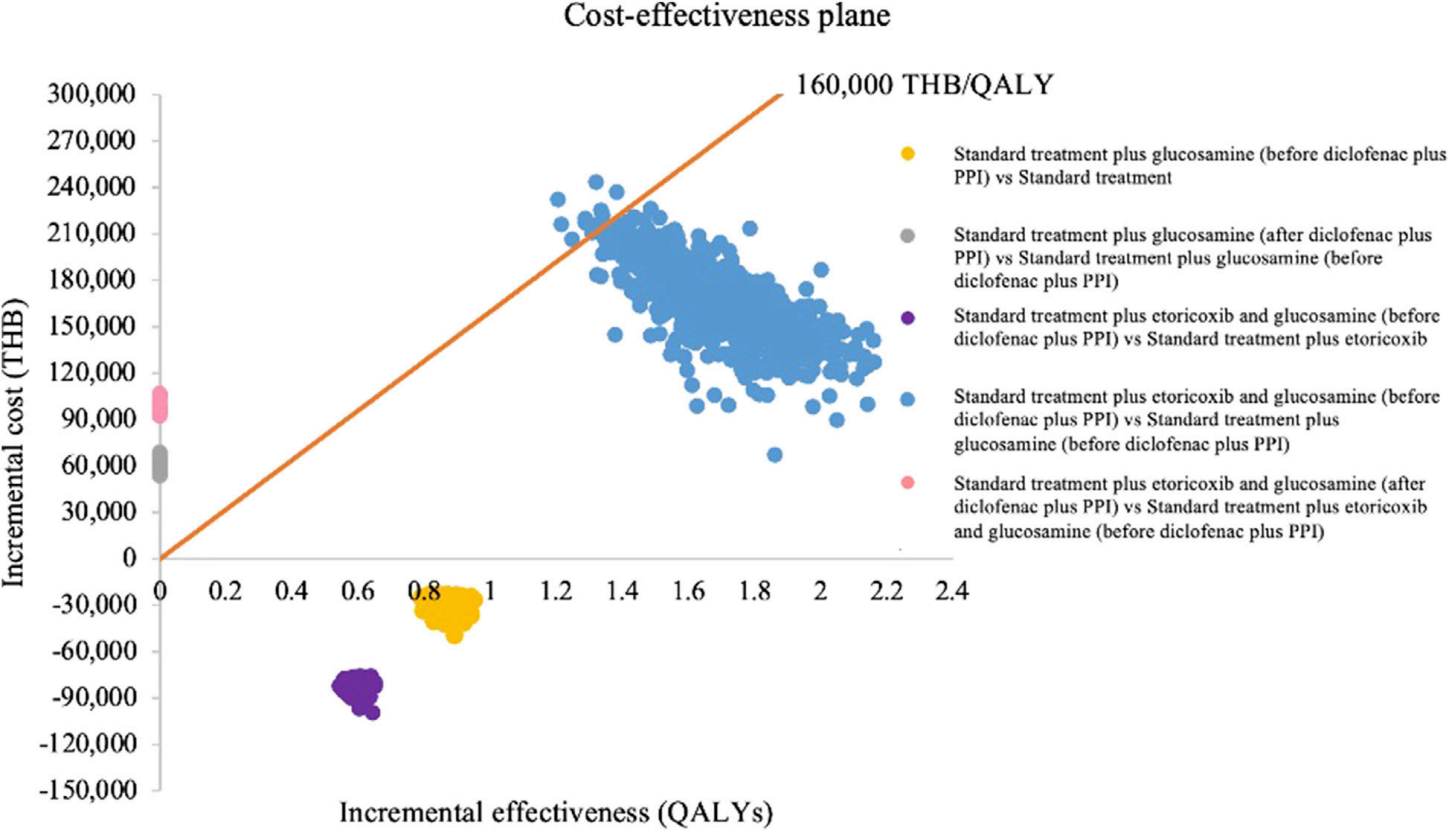
Incremental cost-effectiveness plane. PPI, proton pump inhibitors; QALYs, quality-adjusted life-years; THB, Thai Baht.

**FIGURE 4:**
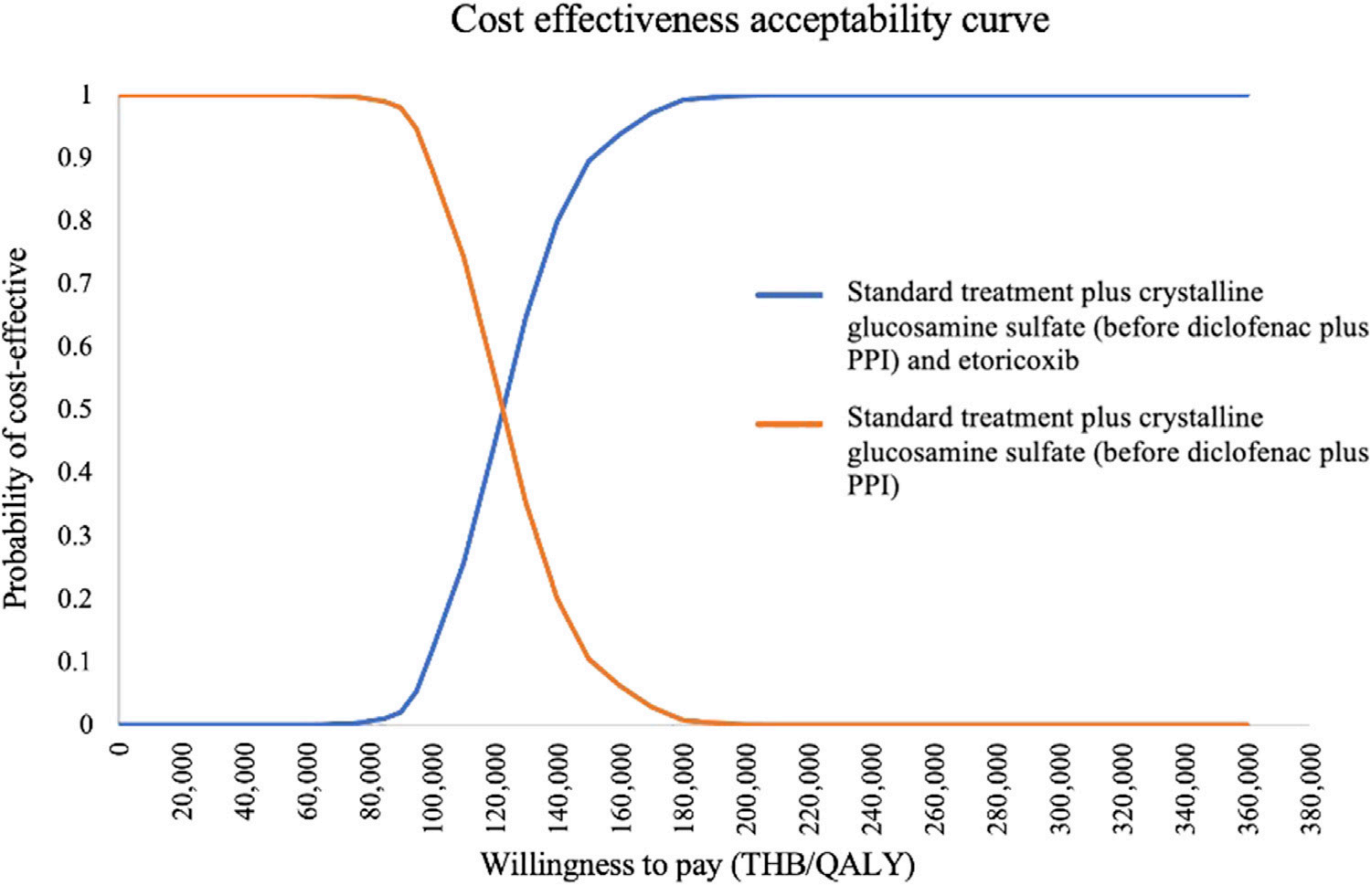
Cost-effectiveness acceptablity curve for standard treatment plus crystalline glucosamine sulfate (before diclofenac plus PPI) and etoricoxib vs. standard treatment plus crystalline glucosamine sulfate (before diclofenac plus PPI). PPI, proton pump inhibitors; QALYs, quality-adjusted life-years; THB, Thai Baht.

The probabilistic sensitivity analysis of 1,000 Monte Carlo simulations was shown in the incremental cost-effectiveness plane (Figure 3). At a WTP of 160,000 THB/QALY, a WTP threshold in Thailand (Tanvejsilp et al., 2019), addition of both crystalline glucosamine sulfate (before diclofenac plus PPI state) and etoricoxib had approximately a 98.8% chance of being cost-effective when compared to addition of crystalline glucosamine sulfate alone. When adding crystalline glucosamine sulfate (before diclofenac plus PPI state) into the standard care sequence, all 1,000 iterations of ICERs fell in the lower-right quadrant, demonstrating that crystalline glucosamine sulfate treatment incurred lower costs and improved QALYs. CEAC was indicated to access the impact of WTP on the probability of cost-effectiveness. The addition of crystalline glucosamine sulfate (before diclofenac plus PPI state) and etoricoxib was cost-effective when compared to adding crystalline glucosamine sulfate alone at the WTP threshold at least 125,547THB (3,472 USD), as shown in Figure 4.

## Discussion

To finding the most efficient alternative of knee OA treatment, including pain relief and adverse events into an analysis is crucial. Our Markov model evaluated the cost-effectiveness of standard treatment compared to adding either crystalline glucosamine sulfate or etoricoxib or both and switching care sequence. As the WTP threshold of 160,000 THB, the results indicated that adding crystalline glucosamine sulfate (before diclofenac plus PPI) and etoricoxib into standard treatment was cost-effective. It is also interesting to note that early initiation of crystalline glucosamine sulfate would be less costly and more effective than delayed treatment or the use of standard treatment alone.

According to our all analysis, the order of care sequence had an impact on the patient’s treatment outcome. One reason might be that crystalline glucosamine sulfate has been proposed as one of the treatment choices for treating mild to moderate knee OA. It affected efficacy in terms of clinical improvement, retardation of disease progression, and decreased risk of undergoing total joint replacement surgery while adverse events were lower than other treatments (Bruyère et al., 2016). These effects when taken into consideration resulted in making an early initiation of crystalline glucosamine sulfate before the use of NSAIDs a dominant strategy.

Recent cost-effectiveness models for etoricoxib treating knee OA have been founded in two studies. Moore A et al. (Moore et al., 2004) concluded that etoricoxib was cost-effective and superior over non-selective NSAIDs plus a PPI or misoprostol. In contrast with another study, Spiegel BMR et al. (Spiegel et al., 2005) reported that non-selective NSAIDs plus a PPI was a dominant strategy and less costly than coxib in patients with high risk of GI or CV events. The different results might be due to assumptions and data used in the analysis. Both studies assumed equivalent efficacy and emphasized only adverse events, whereas our analysis considered these two aspects that would have an impact on the cost and outcomes of each treatment alternative.

For glucosamine sulfate, our findings were consistent with two previous studies which concluded that glucosamine sulfate was a cost-effective therapy. Scholtissen et al. (Scholtissen et al., 2010) reported that crystalline glucosamine sulfate was a highly cost-effective dominant over paracetamol and placebo. Bruyere et al. (Bruyère et al., 2019) showed that crystalline glucosamine sulfate was cost-effective compared to placebo. Inconsistency with two studies’ findings, Black et al. (Black et al., 2009) concluded that glucosamine presented some clinical effectiveness in the treatment of knee OA but not clearly demonstrated about cost-effectiveness which related to the magnitude and duration of quality of life gain. Chaiyakunapruk N et al. (Chaiyakunapruk et al., 2010) estimated the cost-effectiveness of glucosamine sulfate compared with current care and concluded that glucosamine sulfate may not be cost-effective. The contradictory results might be due to the difference of the state model, perspective, and focusing only on efficacy. To the best of our knowledge, there has been no published study evaluating the cost-effectiveness of adding crystalline glucosamine sulfate and etoricoxib into standard treatment compared to standard treatment alone.

Currently, glucosamine sulfate formulation was the only licensed product available in Thailand. It was classified as a nonessential drug (NEDs) and authorized to reimburse in Civil Servant Medical Benefit Scheme (CSMBS) with restriction under the reimbursement protocol (Tantivess and Tangcharoensathien, 2016), whereas other national health insurance schemes, universal Coverage Scheme (UCS), and Social Security Scheme (SSS), did not cover. Consequently, access to crystalline glucosamine sulfate may be limited. According to the results of this study, early initiation of crystalline glucosamine sulfate into the standard treatment is a good alternative to enhance the health outcomes of knee OA patients. This strategy might be used as one evidence integrate with clinical outcome evidence to support policymakers in their decision process of the reimbursement protocol development so that patients can access the appropriate treatment available equally when needed.

There were some limitations of this study. First, combining multiple published data sources from other countries were used when data was unavailable in Thailand. There were probability estimates of pain relief, adverse events, and disutility which might incompatible real situations in Thailand. Second, all cost data were collected in Thailand, even so, the data of adverse events-related costs was published many years later. Finally, our model did not cover all real-world knee OA treatments. We had to select the most appropriate treatment and feasibility to represent the model. Therefore, we recommend increasing all possible treatments and adverse events for further study.

In conclusion, the addition of crystalline glucosamine sulfate and etoricoxib into standard knee OA treatment were cost-effective at the WTP threshold in Thailand. In addition, early initiation of crystalline glucosamine sulfate would be less costly and more effective than delayed treatment or the use of standard treatment alone.

## Data Availability

The original contributions presented in the study are included in the article/Supplementary Material; further inquiries can be directed to the corresponding author.
